# Literature-based discovery: addressing the issue of the subpar evaluation methodology

**DOI:** 10.1093/bioinformatics/btad090

**Published:** 2023-02-14

**Authors:** Erwan Moreau

**Affiliations:** Adapt Centre, Trinity College Dublin, Dublin, Ireland

## 1 Introduction

Nowadays virtually any biomedical research work is available almost instantly in digital form, but exploring the literature is made challenging by the ever-increasing amount of publications. Literature-based discovery (LBD) aims to automatically extract new insights from the scientific literature (Henry and McInnes, 2017). Thus LBD is intended to assist researchers in identifying potentially interesting relations between concepts, potentially contributing to faster and broader scientific progress.

Swanson (1986a) introduced LBD by linking dietary fish oil and Raynaud’s syndrome, noting that both concepts have a known relation to blood circulation. This promising first discovery was quickly followed by another one linking migraine and magnesium (Swanson, 1988) (while the relevance of LBD was established through these two initial discoveries, it is worth noting that these specific conditions were not picked by coincidence, as Swanson suffered from these himself (Smalheiser, 2017); from the point of view of the protocol scientific neutrality, one may question to what extent his personal motivation contributed to these discoveries attributed to LBD). These two initial discoveries are the most commonly used as benchmark for the evaluation of LBD systems (Thilakaratne *et al.*, 2019), sometimes together with a few additional discoveries (e.g. Crichton *et al.*, 2020; Pyysalo *et al.*, 2019). Thus LBD evaluation has been mostly relying on the same small set of discoveries as benchmark for the past three decades. Consciously or not, LBD practitioners can be influenced by their knowledge of the target relations (in clinical trials, double-blind experiments prevent both the patient and the researcher from influencing the outcome). Moreover, a methodologically solid evaluation requires a large and diverse set of target discoveries in order to satisfy the condition of statistical representativity, and consequently to ensure that the results can be generalized to other discoveries.

The author argues that the field is built on sand due to a lack of appropriate evaluation method. Although the evaluation issue is known for twenty years, so far it did not cause any impetus to solve or alleviate it. It does not stop novel contributions to be accepted in reputable journals, despite their often poor evaluation methodology. In fact, the evaluation issue seems to have been admitted as an idiosyncrasy of the field, something the LBD community has learned to live with. As many authors noted (e.g. Ganiz *et al.*, 2005; Thilakaratne *et al.*, 2019; Yetisgen-Yildiz and Pratt, 2009), evaluating LBD is very challenging due to the nature of the task: there is no direct way to assess whether the relations produced by an LBD system will eventually turn out to be significant discoveries. Poor LBD evaluation methodology might contribute to the lack of uptake by the biomedical research community at large: despite a rich state of the art, LBD is still a mostly theoretical field. The lack of solid evaluation methodology is probably a factor which hinders the dissemination of LBD as a general research tool. Crucially, the lack of rigorous evaluation means that there is still uncertainty about the scientific validity of LBD, or at least about the scope of the task. This makes the field of LBD at risk of being an empty promise: an interesting intellectual hypothesis, but which might go extinct unless some solid evidence is brought to back it up.

## 2 State of the art in LBD evaluation

Both Yetisgen-Yildiz and Pratt (2008) and Thilakaratne *et al.* (2019) provide a detailed review of the existing evaluation methods for LBD. A wide variety of methods have been applied: for example Kostoff *et al.* (2021) manually review the corresponding literature in order to validate the predicted discoveries, a method which does not necessarily apply to every kind of discovery; Smalheiser *et al.* (2006) and Henry *et al.* (2021) rely on large-scale collaborative studies, an approach which leads to convincing results but requires considerable effort and funding; Wren *et al.* (2004) show that the observed/expected ratio of a relationship correlates with its strength, thus heuristics based on statistical observations can also be considered. These evaluation methods all have merits and limitations, but the most commonly used is still the *replication* method: given a known discovery at time *t*, the LBD system is provided with the literature available before time *t* and produces a list (often a ranked list) of relations which represent potential ‘future’ discoveries, i.e. from time *t* onward. The performance of the system is estimated based on how close to the top the target discovery is. While this evaluation method is reasonably sound, LBD systems are usually tested only against a small set of confirmed discoveries which have been previously found by existing LBD methods [traditionally the original discoveries made by Swanson (1986a, 1988)]. As already mentioned by Ganiz *et al.* (2005), there are multiple biases in this evaluation methodology.

Hristovski *et al.* (2001) proposed a new evaluation method for their system, later formalized as a principled methodology by Yetisgen-Yildiz and Pratt (2009) and called *time sliced evaluation* in Thilakaratne *et al.* (2019): given an arbitrary cut-off year *t* and a target term *x*, the co-occurrences of *x* which are found after time *t* but not before *t* are considered as gold standard discoveries. This approach solves most of the problems of the replication method, in particular it avoids any bias due to size or cherry-picking specific discoveries. As opposed to the replication method, it can also take into account positive and negative instances, making it possible to measure false negative cases in the LBD system output. This method was adopted for example by Lever *et al.* (2017).

The *time sliced evaluation* clearly solves the problem of using too few instances for evaluation, as almost every target term has multiple co-occurrences in the literature. However, the use of the set of co-occurences as a proxy for the set of discoveries is a dramatic simplification. In fact, very few co-occurences represent a true discovery and the vast majority of the co-occurrences considered as ‘discoveries’ for the purpose of this method are meaningless or poorly informative. For example, co-occurrences may be due to chance [e.g. *Ebolavirus* (D029043) and *Burnout, Professional* (D002055) cooccur in Medline] or involve at least one very generic term [e.g. *Alzheimer’s Disease* (D000544) and *Elderly* (D000368)].

Thus with the *time sliced evaluation* method a LBD system is evaluated against a very large number of relations, but most of them are noise. While the true discoveries are included in the large amount of co-occurrences interpreted as gold standard, their proportion is unknown and likely low. As a result, the final performance does not reflect the ability of the system to predict insightful discoveries, only its ability to predict co-occurrences, which are often arbitrary. Despite a great methodological improvement over the *replication* method, this method is still not fit for purpose.

## 3 Blind spots in the definition of the LBD task

While LBD is unambiguously a data-driven task, there is no large-scale benchmark dataset available to evaluate and compare LBD methods. Clearly, the field would benefit from such a resource, since it would bridge the gap between the replication method (actual discoveries but too few instances and only LBD-based) and the time sliced evaluation (large number of examples but very noisy with respect to their discovery status). In 2005, Ganiz *et al.* (2005, p.34) already emphasized that the field of LBD needs gold standard benchmarks, and that ‘these difficulties stem from a dearth of research into the theoretical foundations for evaluation of LBD systems’. Despite its maturity, the field is still reluctant to create its own quality benchmarks. This is likely partly due to the lack of work on the definition of a discovery.

As an example of the importance of this question, a previous letter to the editor by Kostoff (2007) demonstrated that three LBD discoveries presented in Yetisgen-Yildiz and Pratt (2006) were not in fact discoveries: although the cases were studied in detail in the paper by the authors and presumably checked by the reviewers as well, the validity of these discoveries was not questioned before Kostoff’s (2007) letter. This illustrates how difficult it is to define the boundaries of the concept of discovery, and in particular that different people might have a different understanding of this concept (e.g. social scientists versus biologists). This makes a generic biomedical benchmark dataset unlikely; it is more likely that several distinct datasets would co-exist, possibly corresponding to different domains. This would help clarifying which LBD system is more suitable for which type of discovery.

Although the question of the nature of discoveries was studied in Davies’s (1989) early work, there has been little or no follow-up in this direction of research (to the author’s knowledge, at least). Davies (1989) is an especially interesting study: clearly it appeared in the context of the initial excitement about LBD, and this probably explains the epistemological nature of this work. Like other works such as Swanson (1986b), the field was not yet focused on technical questions: like many other scientific fields, LBD turned progressively from a stage of ‘creative engineering’ into a more mature field with well-established definitions, e.g. the ABC model. This process clarified the scope of LBD at the cost of strongly narrowing the task: the ABC model has become the only one ever considered nowadays, even though (Swanson, 1986b) considers three types of discoveries [to these Davies (1989) adds two categories]. Reviving this line of research would contribute to progress towards better evaluation methods.

The question of evaluation is crucial in establishing the scientific validity of a field. People may sometimes think of evaluation only as a way to measure and compare performance, but it is important to emphasize that evaluation methods formally define the target task: a consensus on evaluation means that the community agrees on what the task consists of, and therefore that the task is clearly defined. To date, the community appears to accept that applying the replication method on a small sample is not only a valid evaluation method for LBD, it is the main standard. This implies that LBD methods evaluated in this way are not demonstrably generalizable. By contrast, any other task in the area of big data and machine learning (ML) relies on large benchmark datasets for evaluation. In general, data contributions tend to be less valued in the research ecosystem compared to theoretical or technical ones. But in the case of LBD, the difficulty in formally defining what a discovery is makes compiling a high-quality annotated dataset even harder. Given the interpretative nature of the task, tackling a large-scale process of data annotation is a complex and risky endeavour. It is easy to see how the investment/reward ratio does not favour addressing the issue: thanks to the constant progress of ML techniques, it requires less effort and involves less risk to contribute yet another technical improvement. As a result, the literature can answer the question of *how* to do LBD, even though the question of *what* LBD does is not fully clear.

## 4 Suggestions to counter inertia

The field of LBD bypassed the step of establishing a solid evaluation methodology during its development. As the field grew it standardized poor evaluation practices, making it more and more difficult to fix the issue. In the author’s opinion, the crux lies in the editorial bias which makes an evaluation method acceptable as long as the method has been employed in an earlier reputable publication. While the replication method was a reasonable workaround a couple decades ago (when the field was still in its infancy and there was hardly any alternative), this method should not be considered appropriate anymore in the context of a mature field. Nobody considers a clinical study tested on a few patients as conclusive evidence, for good reasons; there is no reason why it should be different with LBD results. At first, the time sliced method, which is a more statistically solid approach published a long time ago, should be enforced. The evaluation threshold for accepting LBD papers in reputable journals/conferences should be progressively raised until it catches up with the standards of other fields.

But in the long term, even the time sliced method is not satisfactory (for the reasons mentioned above). The field has no alternative but to restart the efforts towards a formal definition of the problem, including data and evaluation issues. An editorial policy could voluntarily welcome contributions which address these questions, for example. This might require temporarily lowering the quality threshold in order to accept papers on these specific topics (until the evaluation gap is reduced), because a perfect solution is unlikely to appear overnight; the community may have to progressively improve by trial and error.

The field clearly belongs to the big family of data-driven tasks, nowadays almost exclusively relying on ML methods. Other similar fields rely heavily on benchmark datasets for evaluation, often even spawning new sub-communities devoted to data annotation standards and evaluation measures. For example, Machine Translation conferences/journals regularly have special issues or workshops devoted specifically to these questions (Machine Translation is another highly interpretative task). A common way to marshal a significant community effort around evaluation or data standards is to organize a scientific competition (a.k.a shared task): the organizers design the precise target task(s), they provide the input data and the scoring method, the participants submit their system/entry, which are then evaluated according to the predefined scoring method. Participants are incentivized by the visibility offered to their work, especially if they reach the top of the ranking. This strategy can lead to significant progress, especially if the competition becomes a regular event hosted by a major conference for instance. This is not a new or original idea, but it is likely that the issues highlighted in the previous section prevented this to happen so far.

In any case, the creation of large annotated datasets requires a conscious effort by a community. There must be some funding devoted to designing and implementing the annotation process. There should also be discussions about the annotation methodology, because clear annotation guidelines are needed in order to prevent errors or noise in the labels. Similarly, the question of sampling the initial dataset of candidate discoveries matters. The interpretative nature of the task must be addressed as well in this context. For instance, one may consider a statistical approach to annotation, sometimes used with highly subjective tasks: a fair number of annotators are asked to label the same samples, and the resulting proportion of annotators selecting a particular label is assumed representative of the likelihood of this label. But even in this option, the annotators must be provided with reasonably clear instructions.

It is hard to imagine that any of this can be achieved without strong collaboration across the LBD community (maybe even broader than this, in order to include the biomedical experts that LBD is meant to help). Perhaps the community could establish a scientific society devoted to progress in the field, which could discuss, decide and organize the priorities. For example, the Association for Computational Linguistics, created in 1962, has had a clear impact on the progress of the field of Natural Language Processing and is recognized as a trustable source of standards. This might create a feeling of ownership around a common goal, encouraging individuals to tackle more ambitious projects in LBD.

## 5 Conclusion

In the research ecosystem, continuous progress is made by ‘standing on the shoulders of giants’, i.e. the corpus of past contributions makes new contributions possible. The LBD field has crystallized around its seminal concepts rather than refined and improved on them. In general, contributions questioning and discussing the design and/or the evaluation of a task tend to be a harder sell than contributions proposing new models or technical improvements. A design/evaluation work often emphasizes the limitations of existing (and possibly well established) practices. Moreover, first attempts at redefining a task may not always immediately succeed at finding the best way to formalize the problem. This is why a community has to proactively encourage such work, especially if there is a consensus about a major evaluation issue. It would not be wise to ignore this dangerous flaw in the foundations of the field much longer.

Financial Support: None declared.

*Conflict of Interest*: none declared.
